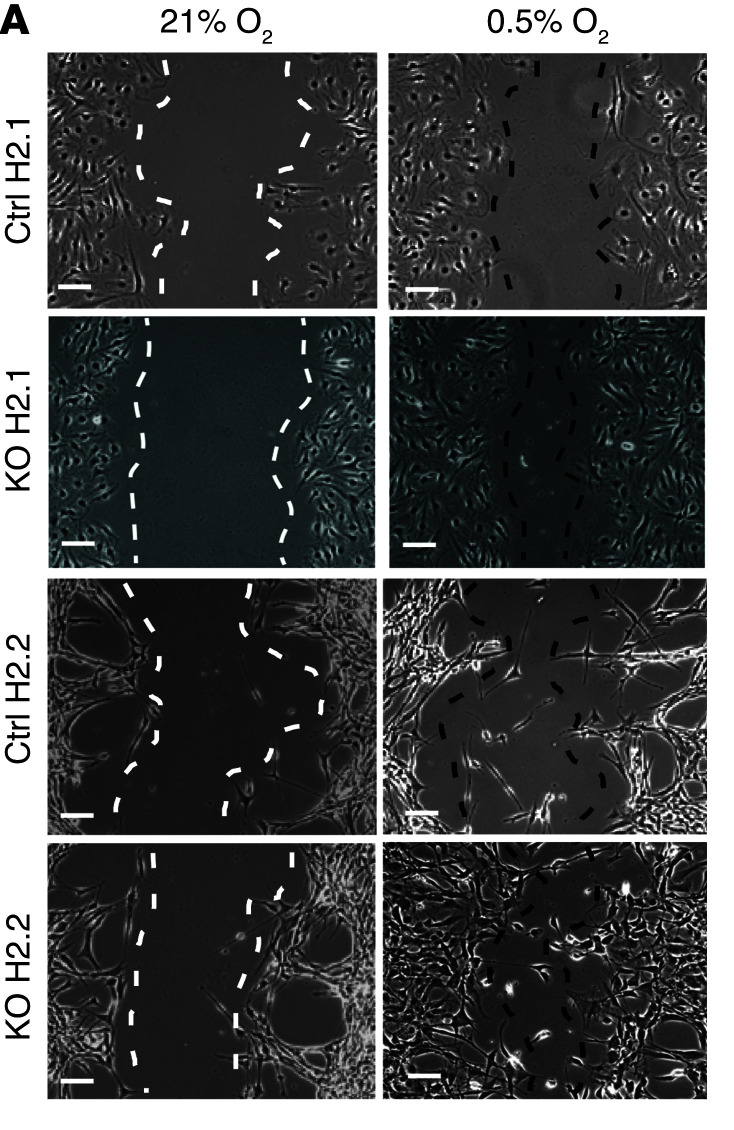# Endothelial HIF-2α regulates murine pathological angiogenesis and revascularization processes

**DOI:** 10.1172/JCI180863

**Published:** 2024-04-15

**Authors:** Nicolas Skuli, Amar J. Majmundar, Bryan L. Krock, Rickson C. Mesquita, Lijoy K. Mathew, Zachary L. Quinn, Anja Runge, Liping Liu, Meeri N. Kim, Jiaming Liang, Steven Schenkel, Arjun G. Yodh, Brian Keith, M. Celeste Simon

Original citation: *J Clin Invest*. 2012;122(4):1427–1443. https://doi.org/10.1172/JCI57322

Citation for this corrigendum: *J Clin Invest*. 2024;134(8):e180863. https://doi.org/10.1172/JCI180863

The authors recently became aware that the image presented in Figure 4A for the 21% O_2_ KO H2.1 sample was a different crop as the image presented in Figure 4A for the 21% O_2_ Ctrl H2.1 sample. In addition, the image presented in Figure 4A for the 0.5% O_2_ KO H2.1. sample was a duplicate of the image presented in Figure 6E for the Ctrl H2.1/0.5% O_2_/DMSO sample. The authors were able to provide the correct images from the original experiment and replaced the 21% O_2_ KO H2.1 and 0.5% O_2_ KO H2.1 in Figure 4A. The correct figure panel is shown below. These corrections do not alter any conclusions of the paper.

In addition, the authors wish to clarify that the images presented in Figure 8A are shown again in Supplemental Figure 10A as part of the full time course of the experiment (day 14 column). The updated legend text for Supplemental Figure 10A appears below:

**Supplemental Figure 10. Dll4 and Dll4/Ang2 are sufficient to reverse endothelial HIF-2α deletion phenotypes in a hindlimb ischemia model.** Blood flow, foot movement score and necrosis were assessed for Ctrl and KO mice after femoral artery ligation and intramuscular injection of an empty viral vector, a viral vector expressing Dll4 or Dll4 vector in combination with intravenous Ang2. Blood flow was quantified by laser Doppler. (**A–B**) Representative images (**A**) obtained by laser Doppler showing the efficiency of the surgery distally to the occlusion side and the progressive blood flow recovery after 21 days for Ctrl and KO mice. Note that images in the day 14 column also appear in Figure 8A. (**B**) Quantitative laser Doppler analysis showing the left-to-right limb ratio after occlusion of the femoral artery at day 7. KO mice display a delayed restoration in perfusion compared to Ctrl mice. However, flow is completely restored for mice injected with a viral vector expressing Dll4 or Dll4 vector in combination with Ang2. (**C**) Foot movement was determined and scored between 0 and 4 as a functional read out parameter to assess flow deficits after ischemia. Active foot movement was significantly impaired in KO mice and restored in the presence of Dll4 vector or Dll4 vector in combination with Ang2. (**D**) Percentage of mice in each group presenting necrosis or amputation was determined. More KO mice had to be euthanized due to amputation or necrosis of the limb. Injection of Dll4 vector or Dll4 vector in combination with Ang2 significantly decreases necrosis and amputation. (Ctrl *n* = 20, KO *n* = 20) Data are means ± SEM. *P < 0.05 and **P < 0.01.

The authors regret the errors.

## Figures and Tables

**Figure:**